# High PI3K/mTOR and low MAPK/JNK activity results in decreased apoptosis and autophagy in nasal polyposis

**DOI:** 10.1016/j.bjorl.2019.12.005

**Published:** 2020-01-15

**Authors:** Fatma Simsek, Erdem Eren, Selen Bahceci, Ibrahim Aladag

**Affiliations:** a Izmir Katip Celebi University, Department of Histology and Embryology, Izmir, Turkey; b Izmir Katip Celebi University, Atatürk Training and Research Hospital, Department of Otorhinolaryngology, Izmir, Turkey

**Keywords:** Nasal polyp, Apoptosis, Autophagy, TUNEL, Eosinophil

## Abstract

**Introduction:**

Nasal polyposis is a progressive inflammatory disease that reduces the quality of life. The role of apoptotic and autophagic pathways in nasal polyposis pathogenesis is not yet clearly known.

**Objective:**

In this study we aimed to investigate apoptotic (MAPK/JNK), anti-apoptotic (PI3K/mTOR) and autophagic (LC3) pathways which are related each other in the nasal polyposis tissues.

**Methods:**

Twenty patients with nasal polyps and fifteen patients going through an inferior turbinate reduction were included in this study. Patients with asthma, Samter triad and allergic fungal sinusitis were excluded from the study. The apoptotic and autophagic pathways were investigated in paraffin-embedded nasal tissue sections of 20 NP and 15 samples from inferior turbinate reduction by H&E and immunohistochemistry with h-score. TUNEL method with apoptotic index was used to demonstrate apoptotic cells.

**Results:**

Decreased immunoreactivity of P38 MAPK (*p* < 0.005) and JNK (*p* < 0.005) were observed in nasal polyposis compared to material from inferior turbinate reduction. This decrease may indicate a downregulation of apoptosis as demonstrated by decreased TUNEL staining in nasal polyposis (*p* < 0.005). The PI3K (*p* < 0.002) and mTOR (*p* < 0.005) immunoreactivities were increased in nasal polyposis. This increase indicates a downregulation of autophagy as demonstrated by decreased LC3 staining in nasal polyposis (*p* < 0.001).

**Conclusion:**

Deficient apoptosis and autophagy through MAPK/JNK and PI3K/mTOR pathways may have a role in the pathogenesis of nasal polyposis.

## Introduction

Nasal polyposis (NPs), benign masses arising mainly from the mucous membranes of the nose and paranasal sinuses, is a common disease with a high recurrence rate.^1^ Despite the significant morbidity of this recurrent disease, central mechanisms regarding the pathogenesis of sinonasal polyposis are complex and poorly understood.

It is assumed that NPs is characterized by morphological changes, such as lining epithelial hyperplasia and metaplasia, inflammatory cells infiltration and stromal fibrosis and edema.^2^ Many cytokines and chemokines are thought to have a major contribution to the pathogenesis of NPs.^3^ For instance, eosinophils are the most common inflammatory cells.^4^ Previous research demonstrated that increased collagen synthesis due to inhibition of apoptosis of eosinophils is a contributing factor for the development of polyps.^5^ In addition, some researchers reported that apoptosis in inflammatory cells is an important factor in the resolution of NPs.^6^ Thus, apoptosis has a role in the removal of unwanted cells associated with NPs. Also, recent studies have suggested that delayed cellular apoptosis has a major role in the pathogenesis of NPs.^7^ In this regard, showing the effective apoptotic pathway might help to prevent the formation of NPs.

On the other hand, autophagy is a highly specific process to mitigate various types of cellular stress. In this process, cytoplasmic contents are sequestered, transported via double-membrane autophagosomes to lysosomes, and degraded. Several pathways are responsible for regulating the apoptosis and autophagy. Mitogen-Activated Protein Kinases (MAPKs) play an important role in the regulation of many cellular processes including cell proliferation, differentiation, and apoptosis. MAPKs consist of the stress-activated MAPKs, c-jun NH2-terminal kinases (JNKs) and p38 MAPKs^8^ that means a low MAPK/JNK activity suppress apoptosis. Conversely, MAPK pathways, the phosphatidylinositol 3-kinase/mammalian target of rapamycin PI3K/mTOR signal transduction pathway, a signaling molecule for protein synthesis and cell survival, is activated and inhibits autophagy,^9^ which means a high PI3K/mTOR activity suppress autophagy.

It has been also reported that the LC3 molecule interacts with the mTOR pathway in autophagosomal membrane formation.^10^ LC3-II is located in the membrane of autophagosomes when autophagy is activated, LC3-II increases directly proportional to the number of autophagosomes.^11^

Autophagy and apoptosis are the important regulators of cell fate. This regulation makes them important in the inflammatory disease process. There is no consensus in the role of these both mechanisms in the pathophysiology of NPs. The purpose of this article is to investigate the role of apoptotic and autophagy-related pathway such as MAPK/JNK, PI3K/mTOR and LC3 in the pathophysiology of NPs.

## Methods

### Patients and tissues

The study was approved by the Izmir Katip Celebi University Atatürk Education and Research Hospital. Informed consent was obtained from each patient, and the study was approved by the ethics committee of the Izmir Katip Celebi University under number 192/13. There were 20 NP and 15 ITR patients in the study and the age range was 18‒55. Also, patients with allergic fungal sinusitis, asthma, AAS intolerance, and Samter triad were not included in the study. The recruited patients did not use systemic or topical steroids, nor did they take other systemic medication at least 4 weeks before the operation. NP samples were collected from patients undergoing endoscopic polyp biopsy for diagnostic purposes and turbinate samples^1^, ^3^, ^12^, ^13^ were collected from patients undergoing turbinate resection because of their compensatory turbinate hypertrophy due to nasal septal deviation.

### H&E and immunohistochemistry

All samples were fixed in 10% formalin for 24 h and processed for embedding in paraffin was using routine protocol. Sections 5 µm thick were cut and stained with Hematoxylin and Eosin (H&E). Also additional sections were used for immunohistochemical staining. Tissue samples were stored at 60 °C overnight and then were deparaffinized by xylene for 30 min. The tissues were then treated with 2% trypsin and incubated in 3% H2O2 solution. Then, sections were incubated with anti p38MAPK (sc-7973, Santa Cruz Biotechnology, Inc.), anti JNK (sc-7345, Santa Cruz Biotechnology, Inc.), anti PI3K (sc-1637, Santa Cruz Biotechnology, Inc.), anti mTOR (sc-8319, Santa Cruz Biotechnology, Inc.) and LC3 (LC3B, NB100-2220, Novus Biologicals. Littleton, CO, USA) primer antibodies in a 1/100 dilution for 18 h at +4 °C. After administration of the secondary antibody, the sections were stained with DAB Substrate system containing diaminobenzidine (DAB-plus subatrate kit, Invitrogen) to detect the immunoreactivity. They were observed blindly with light microscopy (Olympus BX-43, Tokyo, Japan).

Two randomly selected areas were scored, and in sections where all the staining appeared intense, one random field was chosen. At least 100 cells were scored per X40 field for each tissue in all the groups. All sections were scored in a semiquantitative fashion, by considering both the intensity and percentage of cell staining. Intensities were classified as mild (1+), moderate (2+), strong (3+) and very strong (4+). The staining of primer antibodies were graded semiquantitatively and the H-score was calculated using the following equation: H-score = ΣPi (i + 1), where i = intensity of staining with a value of 1, 2, 3 and 4 (mild, moderate or strong, very strong) and Pi is the percentage of stained cells for each intensity, varying from 0 to 100.^14^, ^15^

### TUNEL method

For distribution of apoptotic cells, terminal deoxynucleotide transferase-mediated dUTP nick end-labeling (TUNEL) assay was used according to kit procedure (S7101, ApopTag, Millipore). After all steps,and mounting with Entellan they were examined independently by two histologists and cells, TUNEL-positive or not, were counted on randomly chosen fields per case. Apoptotic index as the percentage of TUNEL-positive cells with positive brown immunostaining was determined. Two observers blinded to treatment assignment assessed the staining scores independently.

### Statistical analysis

The homogeneity of data was assessed using the Shapiro–Wilk test. For normally distributed data, Student’s *t*-test was used; otherwise, we used the ×2 test or the Mann–Whitney *U* test The SPSS software (ver. 16) was used for all statistical analyses. The statistical significance level was set at *p* = 0.05, and 95% confidence intervals were determined.

## Results

### Histology of nasal tissues

**Figure 1:**
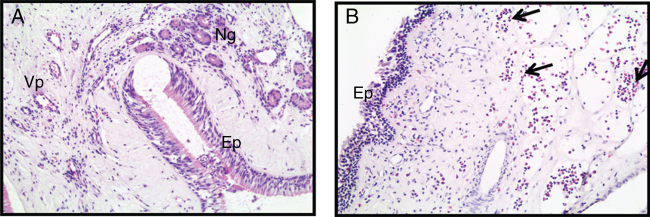
Photomicrographs of hematoxylin and eosin staining within the control (A) and NPs (B) tissues is depicted. (Ep, Epithelial; Vp, Venous plexus; Ng, Nasal glands; Arrow, Eosinophils) (Original magnification ×200).

Hematoxylin and eosin stained samples of nasal tissues are presented in Fig. 1. In samples of turbinate tissue staining is attributed to the submucosal venous plexus and nasal glands (Fig. 1A). NP formation is histologically characterized by submucosal edema and extracellular matrix accumulation (Fig. 1B). Also, in the NP, cell infiltration in the submucosa, especially the eosinophil cells, were significantly higher than in the turbinate tissue where they were seen from epithelial injury (Fig. 1B).

### Immunohistochemical ‒ apoptotic and autofagic ‒ evaluation

Immunohistochemical studies on additional 35 paraffin-embedded nasal tissue sections revealed the localization of the apoptosis related markers and the relationship between their protein expression and NPs tissues.

**Figure 2:**
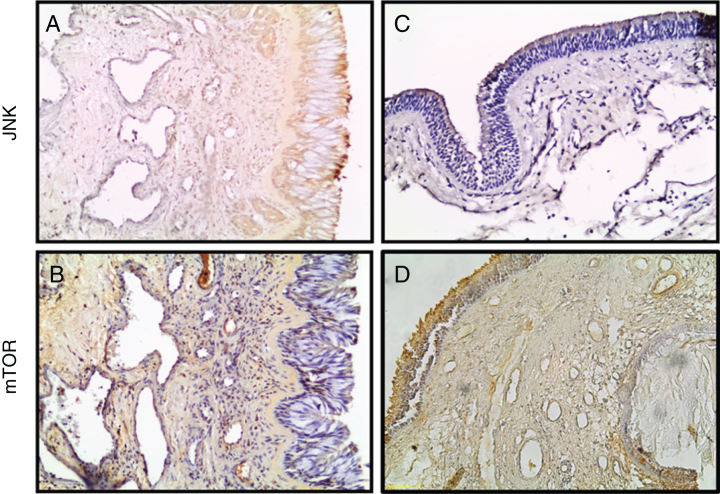
Photomicrographs of JNK, and mTOR staining within the turbinate tissue (A, B) and NPs (C, D) tissues is depicted. (Original magnification ×200).

Positive immunoreactivities were determined in the epithelial and the stromal parts of all the tissues. Examples of immunohistochemical staining are shown in Fig. 2. The anti-JNK immunoreactivity was decreased and mTOR immunoreactivity was increased in NPs (Fig. 2).

For P38 MAPK primary antibody, immunohistochemical staining was moderate, and mild to moderate immunoreactivities of anti-PI3K were found in the NPs.

**Figure 3:**
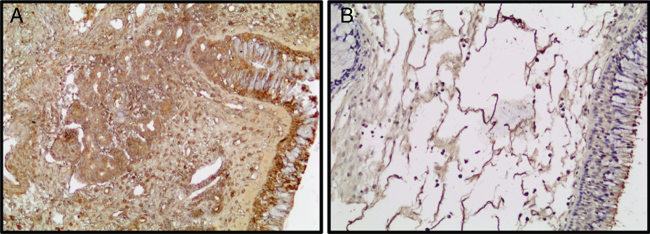
Photomicrographs of LC3 staining within the ITR (A) and NPs (B) tissues is depicted. (Original magnification ×200).

An autophagic marker, LC3 immunoreactivity was decreased in the NPs according to the control group (Fig. 3).

**Figure 4:**
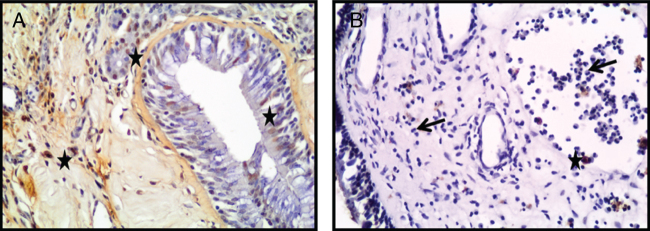
Photomicrographs of TUNEL staining within the turbinate tissue (A) and NPs (B) tissues is depicted. TUNEL-positive cells were seen as brown (star) (Arrow: Eosinophils) (A, B; original magnification ×400).

As a result of terminal deoxynucleotide transferase-mediated dUTP nick end-labeling (TUNEL) staining to assess the presence of apoptotic cells and nuclear DNA fragmentations in the patients, the average of TUNEL-positive cells found was moderate for turbinate tissue (Fig. 4A) and negative to weak for NPs (Fig. 4B). Also, we detected that since the apoptotic cells were fewer in NP tissues, the apoptosis was not in the eosinophils or the other inflammatory cells (Fig. 4B).

The positive staining intensities of P38 MAPK, JNK, PI3K, mTOR, LC3, and TUNEL in the ITR and nasal tissues are summarized in Table 1.

**Table 1:** Percentage of positive staining cells in NPs and Turbinate Tissues (ITR).

	P38 MAPK	JNK	PI3K	mTOR	LC3	TUNEL
NPs	Moderate *p* < 0.005	Weak/Moderate *p* < 0.005	Mild/Moderate *p* < 0.002	Strong *p* < 0.005	Strong *p* < 0.001	Negative/Weak *p* < 0.005
ITR	Strong	Strong	Negative	Moderate	Mild/Moderate	Moderate

**Figure 5:**
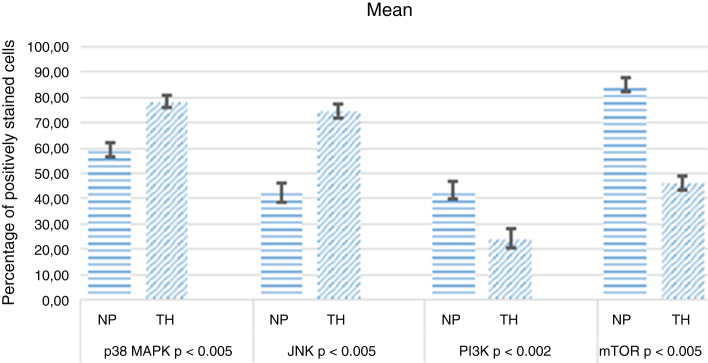
H-score of NPs and ITR samples.

**Figure 6:**
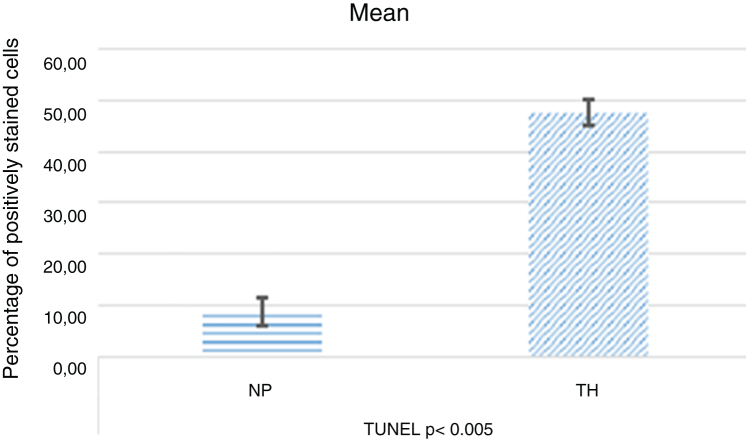
Apoptotic index by TUNEL in NPs and ITR.

The results of the median analysis of the staining are summarized in Figure 5, Figure 6.

## Discussion

In this study, we have demonstrated that decreased expressions of P38/MAPK, JNK and LC3 pathways and increased expressions of PI3K and mTOR pathways may have related to downregulation of apoptosis and autophagy in NPs.

Apoptosis is programmed cell death which useless, irreparably damaged, or unwanted cells are eliminated during development.^16^ Autophagy is a normal physiological process in the body involved in cellular homeostasis and survival mechanisms in normal respiring cells.^17^ While there is little known about the role of autophagy in respiratory illness such as allergic rhinitis and sinusitis,^18^ there is no consensus in the literature on the role of autophagy and apoptosis in the pathophysiology of NP.

Chen et al. studied Light Chain 3 (LC3) protein expression which is a common indicator of autophagy and protein kinase B-mechanistic target of rapamycin (Akt-mTOR) expression in fresh tissue specimens of 5 nasal polyps and 6 normal nasal mucosa specimens.^3^ They found that LC3 expression was decreased and activity of Akt- mTOR increased in NP. Wang et al. also reported decreased autophagy.^19^ In their study, they found that LC3 expression was decreased while COX-2 expression was significantly increased in fresh NP tissues compared with the NM control. Autophagy was deficient in NP tissues and COX-2 is negatively regulated by autophagy in NP-derived fibroblasts.

In another study, Qi et al. suggested that expression of Beclin1 and LC3 expressions in nasal polyp tissue was lower than in inferior turbinate mucosa.^20^

On the contrary, Shun et al. reported that the expression of HIF-1α, LDH, and, LC3II, is increased in nasal polyp specimens. They concluded that analysis of their data indicated that hypoxia may contribute to the formation of the NP by promoting autophagy in NP fibroblasts.^21^ Wang et al. reported that the expression of autophagic proteins LC3 was increased in polyp tissues and the extrinsic apoptosis signaling pathway was remarkably activated in NP epithelial cells.^22^

Küpper et al. investigated the mRNA expression of the apoptosis mediator’s such as caspase 3, 7 and 9; and of p53 protein analyzed by using quantitative reverse transcription-polymerase chain reaction in 25 NPs and 18 control samples. They reported the reduced expression of p53 with caspase 3 and 9 in patients with CRSwNPs (Chronic Rhinosinusitis with NPs) compared those of controls. They concluded that the reduced expression of these apoptosis factors in CRSwNPs might be related to higher proliferation and the perpetuation of inflammatory cells.^23^ In our study, we have found decreased apoptosis and autophagy in NP specimens compared to ITR specimens.

On the other hand, in previous years, the studies showing the role of oxidative stress in the pathogenesis of nasal polyposis are increased.^24^ MAPKs is the major intracellular, oxidative stress-sensitive signal transduction pathway.^25^ Therefore, the expression of MAPK may be one of the key determinants of apoptosis in NPs.

Besides the immunohistochemistry, we investigated the apoptotic cells by TUNEL method. The percentage of TUNEL-positive cells found was decreased in NPs compare to turbinate tissue. Particularly, there was no apoptosis in the eosinophils in NPs tissues. The eosinophil is the main culprit cell in NPs pathology.^26^ In pathological situations, eosinophil production is increased, and these cells migrate into inflamed tissues, where their lifespan is believed to increase by a combination of inflammatory mediators and changes to the local microenvironment.^27^ The removal of eosinophils from the polypoid tissue occurs through apoptosis.^28^

Eosinophils have cytotoxic functions such as releasing major basic protein, eosinophil peroxidase, the eosinophilic cationic protein that causes tissue damage and eosinophilia increases the likelihood of recurrent disease.^29^ Khalmuratova et al. tested the wogonin (5,7-dihydroxy-8-methoxyflavone) in NP and they suggested that wogonin attenuates nasal polyp formation by inducing eosinophil apoptosis.^30^ In another study, the expression of PARP and caspase 8 were increased in bleomycin-treated NP tissue; also the tissues were decreased and then disappeared.^31^

These results show that increased expression of antiapoptotic molecules decreased expression of proapoptotic and autophagic molecules in NPs and especially the absence of apoptosis in eosinophil cells in NPs, may be contributing factor for the development of polyps.

### Limitations

Our study has several limitations. We used immunohistochemical methods instead of gene expression and western blot tests which are quantitative. To decrease the subjectivity of the results two blinded observers evaluated the results as described in the methods.

## Conclusion

Apoptosis, eosinophilia and hyperproliferation are the major cellular processes in nasal polyposis and these proteins may take part and play some important role information of this disease and the targeting of new treatment protocols.

## Funding

This study was supported by the Izmir Katip Çelebi University Scientific Research and Project Coordinatorship (nº 2013-3-TSBP-16).

## Conflicts of interest

The authors declare no conflicts of interest.
